# A Review of Risk Factors of African Swine Fever Incursion in Pig Farming within the European Union Scenario

**DOI:** 10.3390/pathogens10010084

**Published:** 2021-01-19

**Authors:** Silvia Bellini, Gabriele Casadei, Giorgia De Lorenzi, Marco Tamba

**Affiliations:** Istituto Zooprofilattico Sperimentale della Lombardia e dell’Emilia-Romagna, 25124 Brescia, Italy; gabriele.casadei@izsler.it (G.C.); giorgia.delorenzi@izsler.it (G.D.L.); marco.tamba@izsler.it (M.T.)

**Keywords:** African swine fever, pigs, wild boars, pigs farming, European Union, risk factors

## Abstract

African swine fever (ASF) is a notifiable viral disease of pigs and wild boars that could lead to serious economic losses for the entire European pork industry. As no effective treatment or vaccination is available, disease prevention and control rely on strictly enforced biosecurity measures tailored to the specific risk factors of ASF introduction within domestic pig populations. Here, we present a review addressing the risk factors associated with different European pig farming systems in the context of the actual epidemiological scenario. A list of keywords was combined into a Boolean query, “African swine fever” AND (“Risk factors” OR “Transmission” OR “Spread” OR “Pig farming” OR “Pigs” OR “Wild boars”); was run on 4 databases; and resulted in 52 documents of interest being reviewed. Based on our review, each farming system has its own peculiar risk factors: commercial farms, where best practices are already in place, may suffer from unintentional breaches in biosecurity, while backyard and outdoor farms may suffer from poor ASF awareness, sociocultural factors, and contact with wild boars. In the literature selected for our review, human-related activities and behaviours are presented as the main risks, but we also stress the need to implement biosecurity measures also tailored to risks factors that are specific for the different pig farming practices in the European Union (EU).

## 1. Introduction

African Swine Fever virus (ASFV), a large DNA virus belonging to the family Asfaviridae, is one of the most worrying swine pathogens, and its spread into new countries of the EU would lead to devastating and unrecoverable economic losses to the entire swine production sector. Within Europe, the first ASFV incursion was reported in Portugal in 1957. It took until 1995 to officially eradicate the disease from the continent, with the exception of Sardinia island, where ASF is still endemic to this day. In 2007, a new introduction of the highly virulent genotype II of ASFV was reported in Georgia, where pigs were probably fed ASFV-contaminated pork brought in on ships [1]. Following the incursion into Georgia, the disease spread to other countries in the Caucasian region and to the Russian Federation. Despite all the preventive measures put in place since 2007, in early 2014, ASF reached the EU, with the first case reported in Lithuania, followed by other cases in different countries [2,3,4,5]. Since at present there is no veterinary treatment or vaccination option, prevention and control of ASF must rely mainly on biosecurity measures in all those situations where a potential risk of introduction and/or diffusion is identified. To better address preventive and control measures, risk assessments need to be tailored to the targeted disease and to the farming systems in which they are to be implemented, taking into consideration the epidemiology of the disease, the duration of pathogen excretion in infected animals, the main routes of excretion, survival of the pathogen in the environment, and its routes of infection [6,7]. In the EU, pig farming is one of the most significant economic sectors: it represents 8.5% of the EU agricultural industry output (year 2016), 50% of the entire European meat production (year 2018), and 62% of the total meat exports from EU (year 2018) [8]. European pork production is scattered among several types of farms with much variation among Member States. Three quarters of pigs are reared by just 1.5% of the largest fatteners. Small pig producers are mostly found in the 13 Member States that joined the EU since 2004, which creates a decreasing size of the herd [9]. Considering the Working Document of the Directorate General for Health and Food Safety: “African swine fever strategy for Eastern part of the European Union. SANTE/7113/2015-Rev 12” [8], European pig farms are classified into three categories: (1) non-commercial farms (NCF), where pigs are kept only for fattening for own consumption and neither pigs nor any of their products leave the holding; (2) commercial farms (CF), which sell pigs, send pigs to a slaughterhouse, or move pig products off the holding; and (3) outdoor farms, in which pigs are kept temporarily or permanently outdoors. Rather than taking into account the size of the farm or the type of establishment (breeding, fattening, etc.), this classification considers the commercial attitude of the holdings. In this way, it considers the risk of spreading the disease by trading pigs and the risk for the farm of being exposed to an external source of infection, such as the presence of infected wild pigs or soft ticks. In a nutshell, pig farms are categorized on the basis of the risk of ASF spread and, based on that, targeted biosecurity measures have been established. A peculiar subtype of outdoor farming, common in the south-eastern countries of Europe and the Iberian peninsula, is free range farming, a traditional practice in which autochthonous pig breeds are usually reared in extensive or semi-extensive production systems that may facilitate contact between pigs and wild boars, contributing to the possible spread of ASFV [10]. As ASF is a disease transmitted by contact with other infected animals or fomites, outbreaks in domestic pigs have been correlated with wild boar cases, pointing to a link between the risk of ASFV introduction into domestic pig herds and the degree of contamination of the external environment [11]. Moreover, due to its high resistance, it is often difficult to pinpoint ASF primary route of transmission which, in most cases, might be mediated by human activities rather than by contact with wild animals [4,11,12,13,14]. The worldwide spreading of ASFV and its dreaded effects on the agricultural pork production sector have led to a plethora of studies that identified several risk factors deemed responsible for the transmission of ASFV to domestic pig populations: farm visitors, wild boar presence, unlawful behaviours, legal animal and product trade, lack of biosecurity measures, ticks, and other arthropods, just to mention a few [2,15,16,17,18]. However, so far, risk factors have been examined with specific focuses on specific scenarios in several parts of the world [12,13,19,20], but a systematic approach to the ASFV transmission risks associated with domestic pig farming within the EU area is still lacking. The aim of this review is to identify the main risk factors for the spread of ASF in the domestic pig sector, taking into consideration the epidemiological characteristics of the current scenario in the EU and the different pig farming systems. This information can be relevant in assessing the level of risk of individual holdings in order to plan specific preventive measures.

## 2. Results and Discussion

Few analytical studies have been conducted to identify risk factors involved in the introduction and spread of ASF at the farm level. For this review, a total of 52 publications were finally selected and reviewed. Risk factors that can promote the introduction and spread of the virus at the farm level are multiple: poor farming practices and low biosecurity levels [5,7,21,22], swill feeding and slaughtering on the farm [23], introduction of purchased pigs [24] and products, human behaviours and activities, environmental factors, factors related to society, and the cultural background of the farmers [13,21,22,25]. In other cases, factors deemed to increase the risk of outbreaks at the farm level include the density or the size of the herd [26], a free-ranging husbandry system [27], the proximity to an infectious farm [18,28], usage of out-of-farm semen [29], ova or embryos on breeding farms, contact with wild boars and external pigs [2,16], and improper disposal of carcasses and manure [21,22]. The European Food Safety Authority (EFSA), assessing the risk of spread of ASF in south-eastern Europe in 2019, considered the following as the main risk factors for ASF spread in domestic pig populations: swill feeding, the presence of free-ranging pigs in some areas of a country, the presence of a substantially high number of smallholders in the country, and home-slaughtering [10]. Jurado et. al. [2] reviewed 52 documents, and a panel of experts in the field identified 37 preventive measures to prevent ASF introduction and diffusion among domestic pigs. In their effort, the authors described the following risk factors for the three types of farms (commercial, non-commercial, and outdoor): identification of animals and animal movements, swill feeding, contacts among pigs from different farms, and the presence of feral pigs and wild boars. We grouped the risk factors presented by the literature selected within our review process into 7 categories: biosecurity, swill feeding and slaughtering on farm, trading of pigs and products, human activity factors and farm management, sociocultural risk factors, ASF in wild boars as a risk for neighbouring farms, and ticks and other blood feeding arthropods.

### 2.1. Biosecurity

High levels of farm biosecurity are considered the most important tools for preventing ASFV introduction on a farm [7]. Biosecurity measures on farms and especially at the farm entrance (thorough cleaning and disinfection of buildings, transport vehicles, and personal protective equipment) and health and safety regulations on farms have a role in the introduction of ASFV that remains difficult to quantify [12,30]. Lamberga et al. [21,22] recently described an ASF outbreak in a large commercial pig farm in Latvia, where the weakest points identified were the entrances of the farm and the sanitary filters (locker rooms) for the staff that did not provide clear and clean separation between staff reception areas and the animal accommodation. Contaminated fomites and meat or meat products were a potential risk factor for the introduction of ASFV in the herd, considering that farm employees were allowed to bring and eat their own food inside the farm. Additional risk factors identified in the study were contaminated vehicles transporting pigs or carcasses, feed or bedding originating from areas in which wild boars have had access, and the possibility that farm employees or other persons visiting the farm participated in hunting or were otherwise involved in activities linked to wild boars [21,22]. Outdoor-bred pigs and pigs with access to the outside of farm facilities could have contact with the wild boar population. This type of pigs farming, typical of most small backyard farms, represents one of the weakest links in the biosecurity chain and the biggest risk factor for ASF introduction [10,31]. Low biosecurity farms, high density of wild boars, and vector presence are deemed to be the most dangerous combinations for the spread and persistence of ASFV in domestic low biosecurity farming [32]. A different scenario was reported in Romania where, despite the high rate of outbreaks in domestic farms, primarily with low levels of biosecurity, only a few cases of ASF in wild boars were detected [11]. Even if a correlation between domestic pig outbreaks and wild boar cases was considered possible, the most significant contribution to the introduction of ASF into domestic farms was deemed to be the inadequate biosecurity level of small domestic farms and, more precisely, the indirect contact through contaminated fomites or environment [11].

### 2.2. Swill Feeding and Slaughtering on Farm

While swill feeding was banned in the EU in 2002 after the UK Foot-and-Mouth Disease (FMD) epidemic, epidemiological studies of ASF outbreaks [5,18,31,33] have shown that this practice is still used in many countries and that there is a lack of detailed information on the nature, frequency, and distribution of swill usage in the pig production sector. Some European pig herds were infected via the introduction of swill feeding since the long-term survival of the virus in pig meat and animal remains represents an important risk for indirect disease transmission [5,31,33]. In non-commercial farms, swill as a supplementary feed is probably commonly used and ASFV-contaminated pork or pig products could represent a possible source of infection. A study on the risk factors affecting the distribution of ASF in Sardinia after the beginning of the eradication program in 1993 correlated the high number of close farms within a municipality with a higher risk of ASF incursion probably due to a more frequent adoption of swill feeding in small-scale closed farms [34]. Often, the contaminated meat may have been stored chilled, frozen, or treated and may have been kept over long periods of time, thus acting as the main mechanism for ASFV maintenance and reintroduction [3]. Illegal imports of pork and meat products could be a risk factor for ASFV introduction in a farm if in combination with swill feeding, for domestic pigs and also for wild pigs if they have access to food waste [7]. Swill feeding together with illegal movements of infected pork meat, suspected cases underreporting, and “emergency sales” represent important risk factors in the spread of the disease [17,18,35,36,37] as do illegal slaughtering and slaughtering of sick animals [38]. These behaviours are often rooted in the socioeconomic situation of poor farming regions characterized by inadequate understanding of the ASF threat and by disinterest of citizens and owners of animals in ASF eradication due to a misunderstanding of the problem and unwillingness to undertake the costs of any change to their traditional animal farming system. Within these contexts, reduced trust in the veterinary surveillance system and in the official authorities leads to the sale of pigs and pork without veterinary certificates and illegal turnover, while fear of insufficient compensation upon culling leads to emergency slaughtering on farm and failure to disclose early cases of the disease or animal deaths [39,40].

### 2.3. Trading of Pigs and Products

ASF spread has been linked to trade-related factors, with transport implicated in the transmission of the disease as well as pig restocking and pig exchange with neighbouring districts. A spatiotemporal analysis of the outbreaks in the Russian Federation identified a correlation between the distribution of cases among domestic pigs, the main transportation routes, and the population density distribution. Thus, it was hypothesized that the trade of animals and animal products could have a possible causal effect on the disease spread [26,41,42,43,44,45,46,47,48]. Taylor et al. [49] used data from cases of ASF in 2018 within Europe to estimate the prevalence of diseases in the pig population and in wild boars in order to compute the risk of initial infection via 3 potential routes: legal trade of live pigs, legal trade of pig meat products, or wild boar movements. Their results showed that, for Eastern European countries, where numerous cases of ASF had already been reported, movement of wild boars was a high risk of ASFV transmission to domestic pigs, while legal trade of pigs was considered a high risk of introduction of ASF into Western European countries. Among animal movements, illegal trade is a risk factor due to the difficulty in quantifying this type of activity. Costard et al. [50] tried to close this knowledge gap using a semiquantitative mathematical approach based on factors that influence the likelihood of release of contaminated smuggled meat and products, and subsequent exposure of the susceptible population to ASFV. Their results showed that illegal importation of pork and products is a considerable risk factor of ASFV introduction in new countries [50]. More precisely, on a relative risk scale with 6 categories from negligible to very high, France, Italy, Poland, Romania, and Spain scored at high risk of exposure upon ASF release, while Austria, Bulgaria, Germany, Greece, Hungary, Latvia, Lithuania, Portugal, Sweden, and United Kingdom were at a moderate exposure risk [50].

### 2.4. Human Activity Factors and Farm Management

Pejsak et al. [51], after studying the characteristics of the spread of ASF in Poland from February to August 2014, evidenced that the disease spread slowly and that the probability of ASFV dispersion to further areas was associated with human activities, movement, and trade of ASFV-contaminated pork and wild boar meat. Adding to the already mentioned risks associated to swill feeding, emergency slaughtering on farm, legal and illegal introduction of pigs and pork, human activities mainly associated with visits by veterinarians or para-veterinarians may pose an issue. Boklund et al. [18], in their risk analysis of incursion of ASF in Romanian domestic farms, identified the number of professional visitors during the high-risk period to be a significant risk factor for ASF incursion in backyard farms. In Latvia, a study conducted on 32 ASF outbreaks revealed that primary outbreaks were most probably due to contact with wild boars and swill feeding practices, but secondary outbreaks were related to the entrance of visitors previously spotted on other infected farms [5]. Once again, this finding correlates with the low biosecurity level of such types of pig rearing, as already pointed by other experts [7]. As anticipated above, indirect transmission of ASFV via contaminated feed products is possible [52]. Recently, ASF outbreaks were detected in pig herds located in different countries, with thousands of kilometres separating affected herds, and according to European and Asian recent epidemiological analyses, long-distance (transboundary and transcontinental) movement of ASFV through contaminated feed and feed ingredients should be regarded as a suitable route of ASFV spread, especially within ASF-free areas [52]. ASFV in fact can be transmitted orally through the consumption of both liquid and feed, adding to the role of feed in the emergence of this virus in pig populations worldwide [53]. In fact, a study identified the infectious doses of ASFV (Georgia 2007 strain) for liquid and feed [53], and despite the results showing a higher dose for feed than for liquid, the authors hypothesized that the high frequency of exposure and the higher volumes consumed might make feed an important risk factor for transmission in modern pig farming systems. Moreover, considering that feed production is often highly centralized, there might be a high risk that contaminated feedstuff could be disseminated across a high number of pig farms [53]. Additionally, the low infectious dose of ASFV when ingested via liquid consumption should be considered another risk factor of ASFV spreading through water. The results of an epidemiological investigation in Romanian counties, where the outbreaks were registered especially near the Danube river, seem to point in this direction [11]. The EFSA [4] reported that, in Latvia and Lithuania, transmission of the virus to domestic pigs could have happened via fresh grass and seeds contaminated by wild boar faeces containing ASFV. Moreover, a study [5] conducted on ASF in Latvia suggested that fresh grass or crops contaminated by wild boars and used as feed had been a risk factor for ASF occurrence in backyard holdings. In their recent study, Fischer et al. [54] took six different types of ASF-contaminated field crops (wheat, barley, rye, triticale, corn, and peas) and tested the efficacy of drying and heat treatment towards the inactivation of ASFV. They analysed samples for the presence of viral DNA and infectious virus after drying at room temperature or at moderate temperature (between 40 °C and 75 °C). Their results showed that no infectious virus could be detected after drying but that ASF DNA was detected in all samples by PCR. The authors concluded that the risk of ASFV transmission via contaminated crops (incubated for a minimum time of 2 h at room or higher temperature) is probably low, but they underlined that, to minimize the risk of transmission, crops from ASF-affected zones should not be used for pig feeding.

### 2.5. Sociocultural Risk Factors

The importance of social factors in the context of animal disease control is well recognized. Culture and human behaviour can influence the epidemiology of animal diseases. ASF introduction and persistence are correlated with different aspects like social, cultural, and religious factors. Regarding ASF in Sardinia, the association between culture, habits in animal breading, and disease spreading is strong [40]. The risk of ASF maintenance in Sardinia increases in municipalities with higher levels of human deprivation (defined as a lack of goods, services, amenities, and physical environment), reduced educational levels, and low employment [39,40]. In detail, Loi et al. [40] studied indicators for the presence of ASF in municipalities in Sardinia and evidenced that common indicators for ASF presence could be the age and sex of the farmer, a material deprivation index, the number of farms and animals, a micro-criminality index, and the rate of reported thefts [40]. In Sardinia, the disease has been historically associated with free-ranging pig farming, which is rooted in sociocultural factors and traditional practices defining the very consciousness of self in local people [2,16,39,55]. This type of traditional pig farming is mainly practiced in mountainous areas that are economically deprived and with limited outside access [16,39,40]. It has been proposed by several authors that the difficulties associated with removing free-ranging pigs from Sardinia are seeded in the cultural self-identity of the farmers that refuse to change their habits because this would mean losing their cultural identity [16,39,40]. Moreover, illegal trade of products and pigs has been a characteristic of pig production in Sardinia and is regarded as a factor for the persistence of ASF on the island [27]. Chenais et al. [35] reported the peculiar diffusion pattern of actual ASF epidemics in the Russian Federation and Eastern European countries, which is characterized by long-distance spread most probably caused by human-related actions, as said before. Reported human factors are the transport of contaminated meat and products, swill feeding to domestic pigs, or abandoning contaminated leftovers in wild boar environments. While the latter examples show behaviours driven by improper education over the ASF threats, Costard et al. [36] showed that, although increased awareness of smallholder farmers is desirable, their actions are driven by social acceptance, fear of insufficient compensation upon culling, or economic damages due to trade restrictions imposed by local authorities; hence, adequate financial compensation seems to be the real key driver for these producers. All these social and economic motivations may lead farmers and local operators to adopt behaviours that might negatively affect control of the disease [56]. Compliance with the legal requirements laid down by veterinary authorities, in fact, is central to the success of control and eradication efforts, including ASF [10]. Vergne et al. [28] conducted a study in Germany, Bulgaria, and the Russian Federation with the objective of investigating farmers’ and hunters’ attitudes to reporting suspected ASF cases. They showed that most of them were willing to comply with the regulations: 87% of farmers would timely report an ASF suspicion, 52% of hunters would test hunted wild boars for ASF diagnosis, and 83% of them would report found-dead wild boars. Factors associated with farmers’ unwillingness to immediately report suspected cases were the laboratory incapacity to provide a diagnostic result within one week, fear that reporting suspect cases might have adverse effects on their reputation at the local level, and not feeling able to cope with an ASF outbreak by themselves. As for hunters, the main factor was being unaware of a mechanism by which they could report the presence of wild boar carcasses. The current ASF epidemic in Europe recognizes human behaviours as the major cause of long-distance transmission and virus introduction in domestic pig farms [35]; therefore, these human aspects must always be taken into consideration. Moreover, many times in ASF outbreak investigations, the source of introduction cannot be reliably identified and farmers and other epidemiologically relevant actors may perceive it as a justification for avoiding changes in biosecurity behaviour [57] and may not report noncompliance in the application of legal rules.

### 2.6. ASF in Wild Boars as a Risk for Neighbouring Farms

One of the most controversial risk factors always taken into consideration when assessing the possibility of ASF incursion and spread within the domestic pig population is the presence and propagation of the disease in wild boars. ASFV can be efficiently transmitted by direct contact between wild boars and domestic pigs and by environmental contamination [58,59,60]. Wild boars can in fact contribute to the spread of the virus during the infectious phase of the disease, eliminating it into the environment through infected excretions, secretions, carcasses, or contacts with domestic pigs. Wild boars can mix with domestic pigs, particularly in free-range settings or when farm biosecurity is poorly implemented. In times of scarce feed, wild boars are more likely to approach farms. In such situations, the habitats of domestic pigs and wild boars overlap, facilitating the spread of the disease. Similarly, in Sardinia, the persistence of the disease is significantly associated with free-range farming, a difficult practice to remove because of the cultural identity of the island. Unregistered free-range pigs represent an important virus reservoir and may serve as an ASFV link between the wild boar and domestic pig populations, thus facilitating the spread of ASFV and jeopardizing its control [61]. Therefore, the interaction between wild boars and pigs can prolong ASFV circulation, as observed in many outbreaks in Sardinia and in the Russian Federation [55,62]. Many factors may contribute to the risk of introduction and spread of ASF in wild boar populations and at the wildlife–domestic interface, but many knowledge gaps still exist in this field. The more important knowledge gaps are wild boar density, the presence of a suitable habitat for wild boars, the effect of some measures aimed at reducing wild boar population density, wild boar feeding, human involvement, environmental and biological factors, the role played by wild boar carcasses, and the presence of ASF asymptomatic forms as well as survivors. The EFSA, assessing the risk of spread of ASF in south-eastern Europe in 2019 [10], considered the average wild boar density and the presence of a suitable habitat as the main risk factors for ASF spread in wild boar populations. In EU Member States, wild boars seem to play the main role in ASF infection spread and maintenance, not only for wildlife but also for the domestic pig sector [35]. This seems to depend largely on the population density of wild boars and their interaction with low-biosecurity pig production sites (free-ranging and scavenging pigs in particular). Smietanka et al. [63] evaluated ASF spread in Poland for 18 months between February 2014 and August 2015 and evidenced that the number of cases in domestic pigs were positively correlated with wild boar density. Carcasses of infected animals and food waste containing infected pork products are also thought to be involved [64]. Cadenas-Fernández et al. [65] monitored the interactions between free-ranging pigs and wild boards in an ASF-endemic area of Sardinia. The authors observed that the majority of indirect interactions involved animals that were in movement, suggesting that wild boars and free-ranging pigs do not share resting areas. The authors also reported that indirect interactions were much more frequent near water sources. The authors concluded that free-ranging pigs can act as a bridge in transmitting ASFV between wild boars and domestic pigs, especially in extensive pig production systems [65]. It should be noted though that not all the authors agree in the fact that wild boar density seems to be an important risk factor for ASF spread in the wild boar population [66]. The EFSA [11] evidenced that, from field observations, no wild boar density threshold seems to exist for ASF transmission. ASF outbreaks have occurred and have been reported also in areas with very low wild boar density. The EFSA [11] observed that there are significant knowledge gaps about ASF transmission routes and epidemiology in wild boars and possibly many drivers may determine whether ASF can be sustained in an ecological setting. These may include several factors related to small-scale social structure of wild boars, animal-to-animal transmission, transmission from contaminated environments or infected carcasses, and the role of mechanical vectors in the ASF epidemiology [11]. Moreover, epidemiological analyses conducted by the EFSA on ASF in the Baltic countries and Poland in 2014–2016 did not evidence wild boar density as a potential risk factor associated with the presence of ASF in a region for the countries under study [31,33]. Nevertheless, transmission of the disease in domestic pig farms in the Baltic countries and Poland was related mainly to the epidemic occurring in the wild boar population, the wild boar habitat suitability, and the neighbouring distance from infected wild boars and domestic pigs [15,67]. On the contrary, Croft et al. [68] developed a model to evaluate the possible introduction and spread of ASFV in a wild boar population in England, and their results suggested a relationship between animal density and the rate of disease spread and that the extent of the wildlife–host distribution could be an important factor predicting the duration of an outbreak. Pautenius et al. [69], after studying the spatiotemporal distribution of ASF outbreaks in Lithuania, concluded that there is no correlation between the population density of wild boars and ASFV prevalence in a given region but that it might have an effect on the risk of ASFV introduction into another wild boar population in the case of an increased dispersal distance of adult males. Maintaining low wild boar population levels might prevent long-range dispersals of adult males [69]. Epidemiological analyses conducted by the EFSA on ASF in the Baltic countries and Poland in 2014–2016 showed that the environmental and biological risk factors potentially involved in the occurrence of ASFV in the wild boar population were: the number of settlements, the human population size, the number of domestic pigs, the number of pig farms, roads, and forest cover percentage. The association of ASF presence with human population size, domestic pigs, and pig farms might be an indicator of an involvement of humans in the spread of the disease or could be explained by a higher probability to detect dead wild boars [31,33]. After the introduction of ASFV in Estonia, Latvia, Lithuania, and Poland in 2014, the EFSA [3] analysed the spatiotemporal distribution of notifications and concluded that, from January to September 2014, in several cases, notifications were located too far from each other to be explained by direct contact between animals. This indicated a human involvement in the initial spread of ASFV in the domestic pig population. From September 2014 until March 2015, instead, the expansion of ASF remained local and was mediated by wild boars. The introduction of ASF in Lithuania, in 2014, was linked to infected wild boar movements from the endemic zone in Belarus, while the persistence of the virus in wild boars has been linked to close contact with infected wild boar carcasses [69]. Some authors also observed that the practice of wild boar supplementary feeding could increase the population contact rate and consequently facilitate ASF spread [70]. Also for the EFSA, artificial feeding of wild boars might increase rather than reduce the risk of ASFV spread [4]. EFSA developed a model to identify risk factors for ASF occurrence in wild boars in Estonia using data from 2014 to 2019. The model evidenced that, in Estonia, the probability of finding an ASFV-positive wild boar is directly correlated with the density of pigs farmed in small holdings per local administrative unit (LAU) (animals in small holdings/km^2^). The model also ruled out, as not significant, several other risk factors like average quality of wild boar habitat, average yearly snow depth, average yearly minimum temperature, density of hunters/km^2^, density of hunting dogs/km^2^, density of feeding/baiting places/km^2^, and density of hunted wild boar/km^2^ [17]. ASFV is generally considered extremely resistant, especially if protected by organic material; thus, contaminated wild boar carcasses might facilitate virus persistence for months or even years within a region, significantly influencing the course of an ASF epidemic [35]. Interactions between wild boars and carcasses have been described in several studies based on camera-trapping [71,72] and can represent a serious risk of disease transmission within the wild boar population [72]. Masiulis et al. [73] also studied wild boar behaviours towards domestic pig carcasses disposed in the forest, and they too evidenced that wild boars seem to be more interested in the soil underneath or next to carcasses than to the carcasses themselves. Due to the possibility that carcasses can represent a risk for virus transmission, during the 2017 epidemic in the Czech Republic, public authorities were able to rapidly confine the disease thanks to the great efforts put in place in order to timely find and remove wild boar carcasses. Carcass finding and removal should be done as quickly and effectively as possible, as this type of action is an extremely valid measure for ASF control [35,72,74]. The soil from underneath the carcasses contaminated with ASFV may also play a role in the epidemiology of ASF [35]. Several factors influence the probability that wild boars become infected via direct contact with contaminated soil: the susceptibility of the animals and the type, the frequency, and the intensity of contact. Moreover, considering the short virus excretion phase, the behaviour of wild boars, their ecology, their population density, and the virus resistance in carcasses, ASFV spread through carcasses is considered, for wild boars, a more significant factor than direct contact with live infectious animals [58]. Pepin et al. [75] modelled carcass-based transmission based on data from Eastern Poland. The authors identified carcass-based transmission as the key factor in ASF persistence, especially in low-density host populations where between live animals contact rates were low, and they inferred that contact of a live animal with an infected carcass caused between 53% and 66% of all transmission events.

### 2.7. Ticks and Other Blood Feeding Arthropods

Soft ticks of the genus *Ornithodoros* are the major biological vectors with a significant role in ASFV transmission. They can be infected over long periods of time and act as biological reservoirs of ASFV, allowing the virus to persist locally in the environment [76]. The biological lifecycle of these ticks involves blood-feeding on a host for brief periods of time and then dropping off the host to the ground and either hiding in humid cavities or looking for another host [77]. *Ornithodoros* ticks mainly feed on animals living in burrows or hide in cracks and surfaces that provide sufficient humidity like those found in pigsties in old buildings [78]. Pigs are generally accidental hosts, while wild boars that never rest inside borrows and rarely in the same spot do not seem to be infested by *Ornithodoros* soft ticks. In the Iberian Peninsula, ticks of the *O. erraticus* complex have been reported [77] and their important role in transmission and maintenance of the disease has been demonstrated [78,79]. In Portugal, an outbreak was caused by ticks that harboured the virus for more than 5 years [80]. The presence of ticks was identified as one of the reasons for the longer persistence of the disease in south-west Spain, where extensively farmed pigs could potentially be exposed to *O. erraticus* [78]. *Ornithodoros* ticks have not been implicated yet in the ASF cycle, nor their presence has been clearly demonstrated in other European countries, but information and data about the spatial distribution of *Ornithodoros* ticks are still not detailed. Vial et al. [81] developed a broad scale distribution model for *Ornithodoros* soft ticks in the Western Palearctic region. The Mediterranean region, with hot dry summers and cool winters, as well as the semiarid zones in South-West and Central Asia are highly suitable habitats for *Ornithodoros* soft ticks. Several studies tried to evaluate the presence of antibodies against *Ornithodoros* ticks in the sera of backyard pigs in Sardinia, providing negative results. These field studies suggest that *Ornithodoros* ticks are absent from the island, and consequently, it is assumed that this tick species is not involved in the epizootic cycle of ASF in Sardinia [27,50,79]. Another study did not provide stringent evidence for soft tick–wild boar contact in Germany, so a relevant involvement in the epidemiology of ASF in German wild boars is considered unlikely [60]. In Central Europe and in the Baltic States, hard ticks represent the major group of these parasites, while soft ticks are almost absent [58]. Currently, no study has demonstrated ASFV replication in the organs of hard ticks in Europe. On the other hand, while excluding their role as biological vectors, studies showed that viral DNA can persist in these ticks from 6 to 8 weeks, allowing them to become potential mechanical vectors [82]. Ribeiro et al. [83] showed that *Ornithodoros erraticus* sensu stricto ticks can become competent vectors when feeding on highly viremic pigs and on pigs with lower virus titres (i.e., animals with sub-acute and or chronic infection forms) [83]. Biting flies, such as *Stomoxys calcitrans*, may play a role in the spread of ASFV through their feeding cycle (via bites) [84,85] or by oral ingestion of infected flies [86,87]. ASFV was transmitted to susceptible pigs by *S. calcitrans* infected one hour and 24 h before, and the virus survived in those flies for at least two days without apparent loss of titre [84]. The possibility of transmission through stable flies is also supported by the persistence of high viral titres in these arthropods for up to two days [88]. Ingestion of blood-fed flies could be one possible route of transmission over short distances (e.g., within farms), but it is considered uncommon between wild boars or between pigs within a stable. In a pilot study conducted in an outbreak farm in Estonia, ASFV DNA was detected in small quantities in two samples from flies and mosquitoes. The authors concluded that, even if a role in local transmission cannot be ruled out, the impact of flies and mosquitoes in ASFV local spread is rather low [89]. 

## 3. Materials and Methods

This review aimed to identify factors related to the risk of introduction and spread of ASF in domestic pigs in Europe, as described by scientific publications. The literature search was performed on the 10th of October 2020 using the CAB Abstracts, PubMed, Scopus, and Web of Science databases for scientific articles and publications. A list of key words was combined into a Boolean query to identify titles and/or abstracts of documents of interest. The key words identified as relevant for the search were “African swine fever”, “Risk factors”, “Transmission”, “Spread”, “Pig farming”, “Pigs”, and “Wild boars”. Based on these search terms, the following search string was generated: “African swine fever” AND (“Risk factors” OR “Transmission” OR “Spread” OR “Pig farming” OR “Pigs” OR “Wild boars”). A schematic representation of the search and the selection/exclusion process is reported in Table 1. These searches generated 205 hits for CAB Abstracts, 927 hits for PubMed, 1367 hits for Scopus, and 942 hits for Web of Science, for a total of 3441 records. Hits for each single database were separately imported into Rayyan QCRI [90] for further filtering and selection handling. In the first step of the exclusion process, articles were removed based on the following criteria:

(1) published before 1970;
(2) full article not written in English, Italian, or Spanish;
(3) not related to the European Union scenario; or
(4) abstract not available or conference proceedings.

A total of 994 hits were removed after this first round of exclusion criteria. The remaining 2447 records underwent a second step of processing done by reading the title and abstract of each hit and by excluding all those that were deemed not related to the theme of this review. Another 1963 records were discarded, and the remaining hits (484) were pulled together and screened for duplicates, leaving 285 publications. In the last step of handling, abstracts and/or full texts of each record were read, and the following exclusion criteria were applied:

(1) reviews;
(2) not original data or study;
(3) in vitro study;
(4) cell-level study; and
(5) not containing information on risk factors for farms.

Scientific opinions and expert opinion elicitations were included when judged relevant to the subject of this review, for a total of 50 remaining papers. Two additional publications were found through scanning and reading the 50 selected works, resulting in a final number of 52 records included in this review.

## 4. Conclusions

ASF is a serious, notifiable viral disease of domestic pigs and wild boars that could lead to devastating losses for the entire European pork industry. The current epidemiological situation in the eastern part of Europe represents a constant threat to the EU livestock sector, particularly if the infection pressure remains high at the eastern borders of the EU. No vaccine is currently available to prevent ASF infection; hence, primary prevention measures have a key role in the control strategy of the disease. Our work reviewed the main risk factors involved in the introduction and spread of ASF in the EU as this information can be relevant in assessing the level of risk of individual holdings in order to plan specific preventive measures. The search framework considered the epidemiological characteristics of the current scenario in the EU and took into account the different pig farming systems. Indeed, pig production in the EU is highly heterogeneous regarding farm type (industrialised, outdoor, or backyard), biosecurity standards, and purpose (commercial and own consumption), and the risk of exposure to ASF depends on the country, area and farm location, and the epidemiological situation of the territory. A recent survey on biosecurity implementation was conducted by the World Organisation for Animal Health (OIE) with the aim of examining the way biosecurity is applied across Europe. The survey covered the main fields related to implementation of biosecurity in countries to analyse current strengths and weaknesses and to identify best practices [6]. The study revealed that biosecurity is mostly implemented on farmed animals, with poultry and pigs being the farming sectors with most frequent application of biosecurity practices. Most likely, this is linked to the recent epidemics of avian influenza and ASF in Europe. Additionally, it showed that biosecurity is normally targeted at commercial holdings; however, even other holdings that have access to markets, for example for trade, should be included in the biosecurity programme. Although non-commercial farms can be a dead end in terms of disease spread, backyard units that sell animals at the local or regional levels can have a role in the spread of diseases. The respondents also highlighted the necessity of having mitigation measures to prevent disease spread from wild to domestic animals, and this is particularly relevant for outdoor farming systems [6]. Based on our review of the potential risk factors of introduction and spread of ASFV in pigs farming in Europe, we could conclude that different types of risks affect different types of farming systems, and they need to be specifically considered when preparing a biosecurity program. 

## Figures and Tables

**Table 1:** Search and selection criteria used to compile available evidence for risk factors associated with the introduction of African swine fever (ASF) in the European farming system.

	Cabs Abstracts	Pubmed	Scopus	Web of Science	Total
Primary Search results	205	927	1367	942	3441
Published before 1970; full article not written in English, Italian, or Spanish; not related to the European Union scenario; abstract not available or conference proceedings	135	223	394	242	994
Deemed not related to the theme of the review	49	575	761	578	1963
Remaining records	484				
Duplicates	199				
Not original data or study; in vitro study; not containing information on risk factors for farms; cell-level study; reviews	233				
Remaining records	50				
Final records included in this review	52				
